# Accurate Modeling of Ejection Fraction and Stroke Volume With Mobile Phone Auscultation: Prospective Case-Control Study

**DOI:** 10.2196/57111

**Published:** 2024-06-26

**Authors:** Martin Huecker, Craig Schutzman, Joshua French, Karim El-Kersh, Shahab Ghafghazi, Ravi Desai, Daniel Frick, Jarred Jeremy Thomas

**Affiliations:** 1 Department of Emergency Medicine University of Louisville Louisville, KY United States; 2 Department of Pulmonary and Critical Care Medicine The University of Arizona Phoenix, AZ United States; 3 Lehigh Valley Health Network Cardiology and Critical Care Allentown, PA United States

**Keywords:** ejection fraction, stroke volume, auscultation, digital health, telehealth, acoustic recording, acoustic recordings, acoustic, mHealth, mobile health, mobile phone, mobile phones, heart failure, heart, cardiac, cardiology, health care costs, audio, echocardiographic, echocardiogram, ultrasonography, echocardiography, accuracy, monitoring, telemonitoring, recording, recordings, ejection, machine learning, algorithm, algorithms

## Abstract

**Background:**

Heart failure (HF) contributes greatly to morbidity, mortality, and health care costs worldwide. Hospital readmission rates are tracked closely and determine federal reimbursement dollars. No current modality or technology allows for accurate measurement of relevant HF parameters in ambulatory, rural, or underserved settings. This limits the use of telehealth to diagnose or monitor HF in ambulatory patients.

**Objective:**

This study describes a novel HF diagnostic technology using audio recordings from a standard mobile phone.

**Methods:**

This prospective study of acoustic microphone recordings enrolled convenience samples of patients from 2 different clinical sites in 2 separate areas of the United States. Recordings were obtained at the aortic (second intercostal) site with the patient sitting upright. The team used recordings to create predictive algorithms using physics-based (not neural networks) models. The analysis matched mobile phone acoustic data to ejection fraction (EF) and stroke volume (SV) as evaluated by echocardiograms. Using the physics-based approach to determine features eliminates the need for neural networks and overfitting strategies entirely, potentially offering advantages in data efficiency, model stability, regulatory visibility, and physical insightfulness.

**Results:**

Recordings were obtained from 113 participants. No recordings were excluded due to background noise or for any other reason. Participants had diverse racial backgrounds and body surface areas. Reliable echocardiogram data were available for EF from 113 patients and for SV from 65 patients. The mean age of the EF cohort was 66.3 (SD 13.3) years, with female patients comprising 38.3% (43/113) of the group. Using an EF cutoff of ≤40% versus >40%, the model (using 4 features) had an area under the receiver operating curve (AUROC) of 0.955, sensitivity of 0.952, specificity of 0.958, and accuracy of 0.956. The mean age of the SV cohort was 65.5 (SD 12.7) years, with female patients comprising 34% (38/65) of the group. Using a clinically relevant SV cutoff of <50 mL versus >50 mL, the model (using 3 features) had an AUROC of 0.922, sensitivity of 1.000, specificity of 0.844, and accuracy of 0.923. Acoustics frequencies associated with SV were observed to be higher than those associated with EF and, therefore, were less likely to pass through the tissue without distortion.

**Conclusions:**

This work describes the use of mobile phone auscultation recordings obtained with unaltered cellular microphones. The analysis reproduced the estimates of EF and SV with impressive accuracy. This technology will be further developed into a mobile app that could bring screening and monitoring of HF to several clinical settings, such as home or telehealth, rural, remote, and underserved areas across the globe. This would bring high-quality diagnostic methods to patients with HF using equipment they already own and in situations where no other diagnostic and monitoring options exist.

## Introduction

Cardiovascular disorders contribute immensely to morbidity and mortality in the United States and worldwide. Heart failure (HF) is defined as “a clinical syndrome with symptoms and/or signs caused by a structural and/or functional cardiac abnormality and corroborated by elevated natriuretic peptide levels and/or objective evidence of pulmonary or systemic congestion” [1]. At least 64.3 million people around the world have HF, with that number expected to increase due to improved health care [2]. HF accounts for 1% to 2% of all hospitalizations in high-income countries and is the top cause of admission for patients older than 65 years of age [2]. The United States spent more than US $30 billion on HF in 2012, with a projected increase to US $69.8 billion by 2030 [2]. The mortality of HF ranges from as low as 2% to 4% per year in those with chronic HF and up to 36.5% in those with acute HF [2]. In the United States, the mandatory federal pay-for-performance Hospital Readmissions Reduction Program targets patients with HF and ties reimbursement to 30-day, all-cause, Medicare, fee-for-service readmissions after initial hospitalization for HF; rates reach as high as 23% in some studies [3].

HF is divided into three categories based on the left ventricular ejection fraction (LVEF): (1) HF with reduced ejection fraction (EF), (2) mildly reduced EF, and (3) preserved EF, with EF ranges of ≤40%, 41% to 49%, and ≥50%, respectively [1,2]. LVEF, the percentage of blood in the left ventricle that exits into the aorta during a cardiac cycle, is determined using various imaging techniques, such as echocardiography, cardiac magnetic resonance imaging, nuclear cardiology, or cardiac catheterization [1,4,5]. Thus, the classification of HF depends on the accurate determination of LVEF using expensive diagnostic methods obtained in outpatient or inpatient settings [6,7]. A study from the United Kingdom found that most new HF cases were diagnosed in inpatient settings despite the presence of symptoms that should have triggered an earlier outpatient evaluation [8]. This is at least partly due to barriers such as the availability of transportation, cost concerns, and access to medical facilities. Millions of potential patients with HF worldwide lack access to even basic medical care and are, therefore, unable to undergo risk assessment for heart disease.

The management of patients diagnosed with HF involves serial testing to detect changes in heart function. The techniques used to measure LVEF and other cardiac parameters (cardiac output, indexed stroke volume, etc) can have significant variability, limiting prognostication and treatment efficacy [5]. Diagnostic tests to determine EF also experience great variability, limiting prognostication and treatment efficacy [5]. Due to the somewhat limiting paradigm of EF categories, more regular use of vital measures such as stroke volume (SV) could delineate patients with HF with more granularity, even having implications for treatment [9]. Telehealth represents a potential mechanism to reduce the rates of 30-day readmission in patients with HF [10]. Patients without access to large hospital systems and diagnostic testing would benefit immensely from a low-cost yet accurate method of determining these parameters. The technology harnessing more than 8 billion global mobile phones could vastly improve health care disparities [11].

This pilot study describes a novel diagnostic technology using audio recordings from a standard mobile phone. Prior publications have sought both invasive and noninvasive means of describing cardiac function, but very few have moved out of research phases to clinical or practical use [12-16]. This study aims to establish a set of markers using complex but reproducible mathematics from mobile phone auscultation data that would enable the determination of EF and SV for HF detection, classification, and monitoring. The goal of this study was to demonstrate the feasibility of creating mobile phone models for the classification of LVEF and SV by matching echocardiographic results to phone recordings.

## Methods

### Settings and Participants

This is a pilot prospective study of convenience samples of patients presenting to 2 hospital systems for cardiac workups. At site 1, an urban academic center in the Southern United States, study personnel obtained recordings from patients who received inpatient clinical evaluation for cardiac disease. All participants had a transthoracic echocardiogram within 30 days. At site 2, a large community clinical site in the Northeastern United States, patients already scheduled for outpatient transthoracic echocardiogram were enrolled at the time of the study, and recordings were obtained at the same time as the echo. To minimize audible confounding, the team excluded patients with mechanical heart valves. Patients were also excluded if they had a positive SARS-CoV-2 test, were younger than 18 years of age, or were pregnant.

### Ethical Considerations

This study was approved by the human participants’ research institutional review boards of the University of Louisville ( number 20.0605) at both clinical sites. Written informed consent was obtained from all participants, with the specification that data obtained would be used for research. Patients had the freedom to withdraw at any time, including after data collection and analysis. Privacy and confidentiality were protected by storing all data in secure, encrypted locations. At all times, only IRB-approved personnel had access to the stored data. The participants did not receive any compensation for participation. Patients were consented after the completion of any urgent or emergent diagnostic testing or treatment and after evaluation by inpatient physician teams, to ensure that the study would not delay necessary evaluation or treatment. The study team did not recommend, order, or perform any testing.

### Data Collection

The research team obtained demographics and clinical information from the electronic medical record at each site. Echocardiography was obtained by a single laboratory at each clinical site. The coders used the EF from the final interpretation of the echocardiogram report. Data from site 1 were uploaded into CardBox (Box Inc), a web-based, encrypted research cloud space. Data from site 2 were uploaded to password-protected Google Drive. Data included demographics and formal echo results, as well as other data such as cardiac catheterization reports, vascular imaging, and primary admission diagnoses. Clinical data were matched to respective (deidentified) sound recording files using unique identification codes. SV estimates were based on the Teichholz method—not because it was preferred, but because it was available on most echocardiogram reports.

### Technology and Analytic Method

In addition to open-source Python (Python Software Foundation)-based software, 2 proprietary software were used in the study. The first is Another Sound Recorder (ASR), a recording app developed by NLL APPS. It allowed all recordings to be made in a standardized format across the various phone brands used in the study. The second is Time Series Dynamics (TSD) software developed by Fleming Scientific. It maps time series observation of systems, such as auscultation and waveform data, into a set of descriptive “features.” The mapping relies entirely on models from “dynamics” which, in physics, is the study of motion resulting from force. These “physics-based” features can then be reduced and used as dependent variables in rigorous statistical modeling. The TSD approach, which intends to preserve physical and mathematical rigor throughout the modeling process, eliminates the need for neural networks and makes it possible to work effectively with smaller data sets [17]. The research team has extensive experience with the TSD approach and is currently using it in analogous respiratory mobile phone auscultation studies funded by the National Institutes of Health (NIH) and the Biomedical Advanced Research and Development Authority (BARDA).

Cardiac auscultation acoustics represent primarily the sounds of hemodynamics, which is the movement of blood resulting from forces applied by the heart and vascular system. In traditional auscultation, providers use these acoustics to make inferences about organ and system functionality. The approach in this paper is analogous, except that the acoustics are mapped by dynamics-based models. The approach also differs from more common machine learning approaches to auscultation data processing that typically rely on some combination of frequency domain, linear stochastics, and neural networks.

The research team hypothesizes that, in the classification of hemodynamics, the use of dynamics-based mapping is domain relevant. It is also consistent with published chaos-based and enthalpic-based views of cardiac function [18,19]. In the approach, thousands of dynamics-based features were extracted from the acoustic recordings by TSD software. Selected features were then matched to echocardiogram findings by simple logistic regression [20]. To maintain statistical rigor by avoiding overfitting, the number of features used in the regression was limited to 4, which represents the minimum number of positive or negative testing cases divided by 10. The 4 features were selected by the maximum entropy method that produced 35 similar combinations that were evaluated separately for best performance. Dimensionality reduction was sufficient to eliminate the need for a validation step.

Developed by Fleming Scientific, this proprietary unpublished technology extracts features found in sound recordings from microphones of unmodified mobile phones. By using models from actual physical acoustics, we created algorithms to match echocardiogram findings. The physics models are designed to describe hemodynamics from the acoustic data, thereby making it possible to classify organ functionality directly. The method produces thousands of candidate features for modeling but uses only a few to avoid overfitting. The features were matched to echocardiogram findings by using logistic regression [20]. The approach eliminates the need for neural networks entirely and offers a more rigorous approach to developing artificial intelligence (AI) software.

The research team obtained audio recordings with an assortment of unmodified, nonencased Android mobile phones including LG and Motorola Trac phones and 2 Samsung Galaxy models. The voice recorder was standardized by using ASR, a free open-source app easily installed on any Android product. Both sites used the following ASR settings: WAV format, similar frame speed, mono recording, and no filters or other settings activated. Recordings took place in settings with moderate background noise, such as emergency department rooms, inpatient rooms, and echocardiography labs.

Study personnel obtained the recordings by pressing the microphone lightly into the patient’s skin to minimize surface noise. The participants underwent a 20-second recording at the aortic valve area (second intercostal space just to the right of the sternum). The participants were not required to hold their breath. Patients could be in any position for recording, but most were sitting or semirecumbent. The phones were capable of capturing frequencies as low as 10 Hz, which are well below the range of human auscultation perception. The phones were kept in a secure location at each site, for use only by study personnel.

Recordings from patients underwent physics-based analysis to create the features for use in modeling. The features would serve as independent variables while the dependent variables were parameters such as LVEF, determined by diagnostic testing during hospitalization. By matching selected features to the gold-standard parameters from established diagnostic procedures, algorithms were created that enable common phones to reproduce the gold-standard parameters.

The goal of the analysis was to demonstrate the feasibility of creating mobile phone algorithms for the classification of LVEF and SV by matching echocardiographic results to the phone recordings.

TSD differs from machine learning–based AI in that its overarching goal is to deduce the best physics-based models for making algorithms, thereby maintaining rigor as much as possible. The hemodynamics deduced in this study are consistent with published chaos-based and enthalpic-based views of cardiac function [18,19]. However, this study was not designed to provide physiological verification of the deduced physics.

TSD’s physics-based approach eliminates the need for neural networks and overfitting strategies entirely, potentially offering advantages in data efficiency, model stability, regulatory visibility, and physical insightfulness [17]. TSD’s use of passive signals rather than active signals differs from echocardiogram and most other gold-standard imaging technologies; it uses an analytical foundation designed to describe dynamics directly.

Although the algorithms are based on physics, evaluating them relies on statistical methods consistent with logistic regression analysis. The algorithms were evaluated for the area under the receiver operating curve (AUROC) using the trapezoidal method. Values >0.9 can be interpreted as “excellent,” whereas values in the range of 0.8-0.9 can be interpreted as “good” [21]. Sensitivity, specificity, and accuracy were also calculated and presented with confusion matrix values per common practice. The validity of features was also verified by *Z* test>2 criteria in addition to the heuristic argument.

## Results

### Study Population

In total, 113 patients were enrolled across 2 sites. No recording had to be excluded from the analysis. However, some echocardiogram reports were excluded because of incomplete or inconsistent reporting of EF (n=2) or SV (n=50). From the recent echocardiogram reports, it was possible to match EF findings in 113 patients and estimated SV in 65 patients. For the 113 patients in the EF cohort, the mean age was 66.3 (SD 13.3) years. The cohort consisted of 61.7% (n=70) male patients and 38.3% (n=43) female patients. Regarding race and ethnicity, 77% (n=87) were White, 20.4% (n=23) were Black, and 2.6% (n=3) were Hispanic or Latino. For the 65 patients in the SV cohort, the mean age was 65.5 (SD 12.7) years. The cohort consisted of 66% (n=43) male patients and 34% (n=22) female patients. Regarding race and ethnicity, 74% (n=48) were White and 26% (n=17) were Black. The EF cohort had a mean BMI of 28.3 (SD 6.323) and a mean body surface area (BSA) of 2.03 (SD 0.273). The SV cohort had a mean BMI of 29.3 (SD 6.561) and a mean BSA of 2.05 (SD 0.272).

### LVEF Results

**Figure 1:**
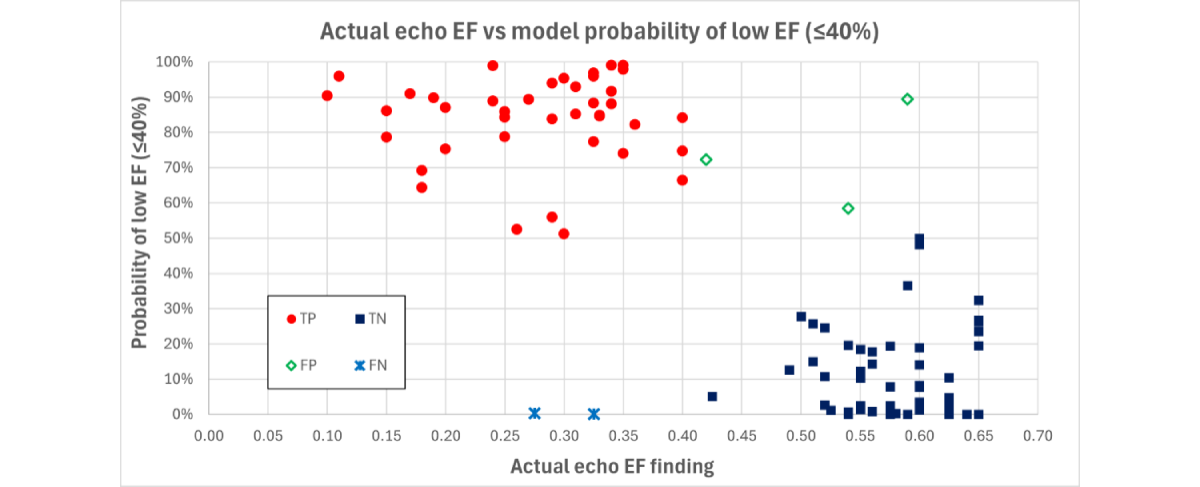
Actual versus predicted ejection fraction (EF). FN: false negative; FP: false positive; TN: true negative; TP: true positive.

The 113-participant EF cohort consisted of 81 participants from site 1 and 32 from site 2. Of note, 57 participants had EF <55% and 56 had an EF>55%. For analysis, the cases were separated into a binary “positive” versus “negative” classification based on the HF disease EF cutoff of 40%. A total of 42 participants with EF ≤40% were designated “positive” in binary classification and they represented 37.2% (n=42) of the cohort; the other 71 (62.8%) participants had EF >40%. The number of features was limited to 4 to avoid overfitting the algorithm. The AUROC was 0.955 (“excellent”), as shown in Table 1. Case separation was also excellent as shown in Figure 1. The EF algorithm accuracy performed similarly across demographics, BSA, and clinical sites (Table 2).

**Table 1:** Ejection fraction algorithm performance and features.

Cases (N=113)	Model evaluation	Features	*Z* test
True negative (n=68)	AUROC^a^ 0.955	1	2.3
False negative (n=2)	Sensitivity 0.952	2	7.2
True positive (n=40)	Specificity 0.958	3	3.9
False positive (n=3)	Accuracy 0.956	4	9.4

^a^AUROC: area under the receiver operating curve.

**Table 2:** EF^a^ model had high accuracy across sex, race, BSA^b^, and age.

Profile			Accuracy
**Sex**			
	Male	0.94	
	Female	0.98	
**Race**			
	White	0.97	
	Black or African American	0.95	
**BSA**			
	BSA<2.04^c^	0.97	
	BSA>2.04^c^	0.95	
**Age (years)**			
	Younger than 66.3^c^	0.98	
	Older than 66.3^c^	0.93	
**Site**			
	1	0.98	
	2	0.97	

^a^EF: ejection fraction.

^b^BSA: body surface area.

^c^EF sample mean.

### SV Results

**Figure 2:**
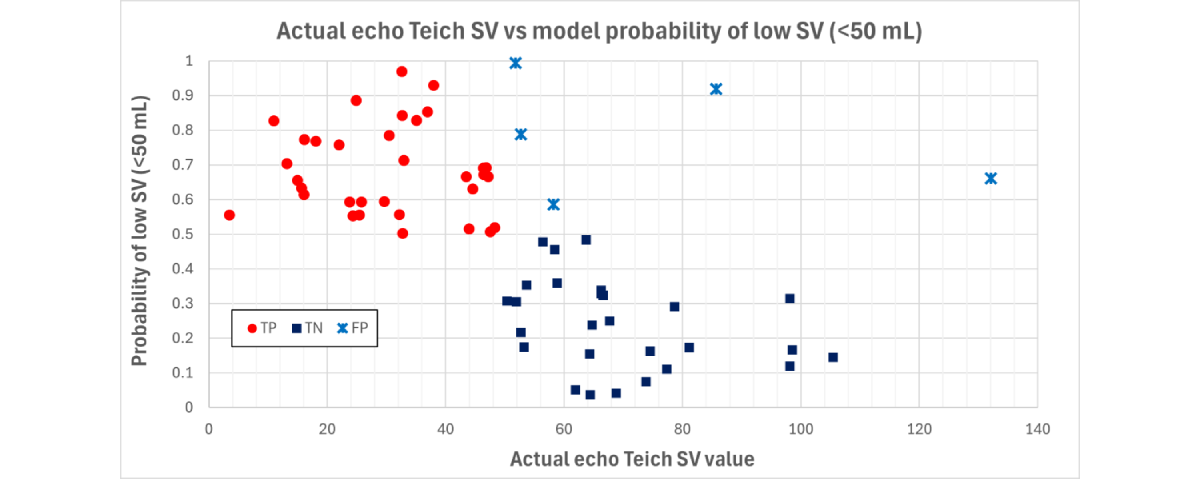
Actual versus predicted SV. FP: false positive; SV: stroke volume; Teich: Teichholz; TN: true negative; TP: true positive.

In all, 65 participants with SV data were all enrolled at site 1. Using a clinically relevant cutoff of <50 mL, 33 (51%) were categorized as positive and 32 (49%) were categorized as negative. For analysis, the number of features was limited to 3 to avoid overfitting the algorithm. Results showed a sensitivity of 100% for the model, with an AUROC of 0.922 (Table 3). Figure 2 illustrates case separation. The SV algorithm accuracy performed similarly across demographics but had a slight drop off in accuracy among patients with higher BSA (Table 4). Acoustics frequencies associated with SV were observed to be higher than those associated with EF and, therefore, were less likely to pass through tissue without distortion.

**Table 3:** SV^a^ algorithm performance and features.

Cases (N=65)	Model evaluation	Features	*Z* test
True negative (n=27)	AUROC^b^ 0.922	1	4.0
False negative (n=0)	Sensitivity 1.000	2	2.5
True positive (n=33)	Specificity 0.844	3	3.1
False positive (n=5)	Accuracy 0.923	N/A^c^	N/A

^a^SV: stroke volume.

^b^AUROC: area under the receiver operating curve.

^c^N/A: not applicable.

**Table 4:** SV^a^ model had high accuracy across sex, race, BSA^b^, and age.

Profile		Accuracy
**Sex**		
	Male	0.93
	Female	0.91
**Race**		
	White	0.92
	Black or African American	0.94
**BSA**		
	BSA<2.05^c^	0.97
	BSA>2.05^c^	0.88
**Age (years)**		
	Younger than 65.5^c^	0.94
	Older than 65.5^c^	0.91
**Site**		
	1	0.92

^a^SV: stroke volume.

^b^BSA: body surface area.

^c^SV sample mean.

## Discussion

### Principal Findings

In this cohort from 2 clinical sites, mobile phone auscultation and dynamics-based modeling allowed accurate detection of low LVEF and SV. These results were obtained using ordinary mobile phones to record from 1 anatomic site with no additional hardware or materials. Prior research suggests that both mobile phones and acoustic recording can assist in HF diagnosis or monitoring; however, no current technologies use basic cellular microphone capability to obtain the acoustic data that can estimate EF or SV. This novel, proprietary unpublished technology has far-reaching potential for screening and management of patients with HF, including the undiagnosed. Perhaps the most obvious use for the technology is telehealth and application to remote and underserved global settings, where even a physical exam by the clinician may not be possible.

Prior work has described technologies that can aid in the monitoring of patients with HF [22-26]. Most technologies use telehealth communications and patient data entry, such as weight, blood pressure, and pulse rate, to risk stratify and monitor disease progress [22,24]. A 2011 Cochrane review established the mortality benefit of telemonitoring in patients with HF [27]. A review by Conway et al [22] identified 4 categories: (1) structured telephone calls; (2) videophone; (3) voice response, which involved the manual input of data using a telephone keypad in response to questions from a computerized voice response system; and (4) telemonitoring. Structured phone calls and telemonitoring showed efficacy in reducing all-cause mortality [22]. Technologies that use true physiologic monitoring require invasive intrathoracic device implantation [28,29], specialized electrocardiography [30], stethoscopes, patches [31,32], or other expensive equipment. Protocols that integrate mobile phones typically use Bluetooth to pair proprietary equipment to a phone in order to transmit data to the care providers [22,25].

Very few described innovations address population screening for HF. A review by Brons et al [33] summarized 99 studies, finding that 100% of algorithms used body weight, 85% used blood pressure, and 61% used heart rate. Bachtiger et al [8] compared 105 patients with low EF to 945 with EF >40% using AI-electrocardiogram (ECG) retrained to interpret a single-lead ECG input. Using a weighted logistic regression from pulmonary and handheld positions, they found an AUROC of 0.91 (95% CI 0.88-0.95), sensitivity of 91.9%, and specificity of 80.2% [8]. One study proposed a method to detect low EF using machine learning or artificial intelligence [34]. Attia et al [35] report on a method using AI-augmented ECG (EKO) to determine the presence of low EF in more than 50,000 patients. The protocol found AUROC, sensitivity, specificity, and accuracy of 0.93%, 86.3%, 85.7%, and 85.7%, respectively. They also found some degree of prediction of future dysfunction: those with a positive AI screen were 4 times more likely to develop ventricular dysfunction in the near future [35].

Shandhi et al [31] compared seismocardiographic data obtained with a wearable sensing patch to objective measurements of pulmonary artery mean pressure and pulmonary capillary wedge pressure following vasodilator infusion during a right heart catheterization, finding reasonable *R*^2^ accuracy (using the Cardiosense technology). These devices use seismocardiological signals in conjunction with ECG signals, thus requiring a hardware device approved by the US Food and Drug Administration (FDA) that must be purchased and maintained. By relying on physics instead of traditional machine learning, a tele-stethoscope does not require the ECG component, making it possible to perform similarly to these more expensive technologies with only an ordinary mobile phone.

One group used computerized acoustic cardiography to detect the third and fourth heart sounds along with systolic time intervals to develop a left ventricular dysfunction index to predict ventricular dysfunction [12]. Their equipment also consisted of an accessory device for a normal ECG machine. Kang et al [36] studied 46 participants to determine the feasibility of phone recordings for detecting heart sounds. Constrained by the presence of 35% of recordings being uninterpretable, the authors found acceptable sensitivity (81%-94%), specificity (79%-100%), positive predictive value (83%-100%), and negative predictive value (82%-92%), with variance depending on which phone was used [36].

Another group tested EF estimation with a novel acoustic-based device (vibration response imaging) that detects low-frequency acoustic signals (10 Hz-70 Hz). The device found sensitivity and specificity around 80%, but the protocol examined requires 36 microphones and a simultaneous ECG [14]. A study using acoustic cardiography in cohorts with and without atrial fibrillation found systolic dysfunction with moderate sensitivity and high specificity (Audicor; Inovise Medical, Inc) [37]. Researchers added sensors to a standard ECG machine to determine 2 systolic parameters: electromechanical activation time and systolic dysfunction index. Another study of the same Audicor device found sensitivity around 80% and specificity in the high 50% range depending on the parameter used [16].

None of these novel approaches show promise for monitoring or diagnosing HF using only mobile phone hardware. Most of the technologies implement proprietary devices and integrate with phones only to transmit data to providers. Tele-stethoscope allows real-time detection of data and rapid transmission of findings directly to clinicians to assist in decision-making. We estimated SV due to its use in approximating cardiac output (SV × heart rate). Noninvasive detection of cardiac output could enhance care for ambulatory and admitted patients. Additionally, SV may represent a parameter that could help distinguish different categories of HF [38].

While this study used research volunteers to obtain the sound recordings, the facile approach allows patients and family members to obtain recordings that can be transmitted with ease using Wi-Fi or cellular signals. This would bring HF diagnosis and monitoring to remote and underserved areas all over the world, to more than 8 billion mobile phones worldwide [11]. Future work will involve matching to other HF diagnostic parameters, such as measures of preserved ejection fraction (early to late diastolic transmitral flow velocity [E/A] to assess diastolic function, and E to early diastolic mitral annular tissue velocity [E/e'] to estimate left ventricular filling pressures) and pulmonary disease markers (spirometry, chronic obstructive pulmonary disease severity scores, and emphysematous changes on computed tomography imaging). In 1 earlier large-scale human study, this technology was used to match phone acoustics to COVID-19 polymerase chain reaction test results to produce a reliable device for disease detection [39].

### Limitations

This work has important limitations. Although relatively small, the sample size was sufficient to demonstrate the feasibility of reproducing echocardiogram EF and SV findings. Additionally, the sample included patients in 2 different cities at 2 different medical centers, 1 inpatient and 1 outpatient. Further studies could center on larger sample sizes and more representative (race, sex, and living areas) recruiting. In a true patient diagnostic model, the best available gold-standard test results, confirmed by diagnosis, would be used rather than echocardiogram reports alone. The enrollment was based on a convenience sample, creating potential selection bias. In the phase 2 study, larger sample sizes will make possible the test or train analysis to demonstrate reproducibility. Larger sample sizes would also make it possible to add more features, if necessary, and reduce the population margin of error.

According to the FDA, a mobile medical app is “a mobile app that incorporates device software functionality that meets the definition of device in section 201(h) of the FD&C Act 11; and either is intended to be used as an accessory to a regulated medical device; or to transform a mobile platform into a regulated medical device” [40]. According to this language, mobile phones and stethoscopes can be considered equivalent. Regarding applicability, the research team views this as a strength rather than a limitation, opening the technology to resource-poor settings all around the world. This would allow fully impromptu data collection in situations where advanced diagnostic equipment is not available and even a physical exam is not possible (telehealth). Phones must be placed directly on the skin and have no motion across the skin, a consideration of importance in future studies where patients will take their measurements. Of note, the fidelity of recordings from this study was not disrupted by background noise; future use in other settings such as ambulance or combat will likely not be limited by ambient noise. Additionally, multiple phone brands were used in the study without any discernible impact on the algorithms.

The comparison of recordings to echocardiogram opens the potential for inaccuracy as transthoracic echocardiogram can have somewhat large margins of error, especially related to EF. The 40% threshold for EF is intended to reduce the rate of false positives. Future work in larger cohorts will allow for a more granular separation of participants. Ongoing work includes recruitment in right heart catheterization and cardiac magnetic resonance imaging patients. Additionally, not all participants at site 1 had the index echocardiogram on the same admission during which acoustic recordings were obtained, but all had the echocardiogram within a 30-day window. Results found no difference in accuracy based on the clinical site or the time of echocardiogram. We did not collect data on the volume status of the participants in the study; acoustic data could potentially vary based on volume status.

It should be noted that no viable features were produced through spectral analysis. One possible explanation is that spectral analysis was unable to manage the nonlinearity of the acoustic signals. Another possible explanation is that it inadvertently created false neighbors among different physical phenomena that happen to share common spectral bands such as low-frequency blood and muscle sounds. Purely from a physics point of view, the features can be interpreted as representing descriptions of fluid and thermodynamics. Although the features used in the modeling are “dynamics-based,” and apparently useful in the modeling, their exact physiological interpretation is unknown. At this stage, all that can be said about these features is that they represent some novel interpretation of hemodynamics as “dynamics.”

### Conclusions

Cardiovascular disease and in particular HF continues to have high morbidity, mortality, and cost worldwide. In this pilot cohort of patients from 2 clinical sites in 2 different cities, passive acoustic recording with mobile phones allowed accurate estimation of EF and SV. No previous study or available technology combines mobile phones and acoustic recording in HF diagnosis or monitoring that could be deployed to low-resource settings. The technology represents a novel and potentially far-reaching tool for the screening and management of patients with known and undiagnosed HF.

## Abbreviations

AI: artificial intelligence
ASR: Another Sound Recorder
AUROC: area under the receiver operating curve
BARDA: Biomedical Advanced Research and Development Authority
BSA: body surface area
ECG: electrocardiogram
EF: ejection fraction
FDA: US Food and Drug Administration
HF: heart failure
IRB: institutional review board
LVEF: left ventricular ejection fraction
NIH: National Institutes of Health
SV: stroke volume
TSD: Time Series Dynamics

## Data Availability

The data sets generated and analyzed during this study are not publicly available due to proprietary analytical techniques. Clinical data are available from the corresponding author on reasonable request.
